# One nerve suffices: A clinically guided nerve ultrasound protocol for the differentiation of multifocal motor neuropathy (MMN) and amyotrophic lateral sclerosis (ALS)

**DOI:** 10.1007/s00415-020-10323-6

**Published:** 2020-12-23

**Authors:** Kai F. Loewenbrück, Robin Werner, René Günther, Markus Dittrich, Robert Klingenberger, Heinz Reichmann, Alexander Storch, Andreas Hermann

**Affiliations:** 1 Department of Neurology, Technische Universität Dresden, Fetscherstrasse 74, 01307 Dresden, Germany; 2 German Center for Neurodegenerative Diseases (DZNE), 01307 Dresden, Germany; 3 Department of Neurology, Elblandkliniken, 01662 Meissen, Germany; 4 Department of Neurology, University of Rostock, 18147 Rostock, Germany; 5 German Center for Neurodegenerative Diseases (DZNE) Rostock/Greifswald, 18147 Rostock, Germany; 6 Department of Neurology, Translational Neurodegeneration Section “Albrecht Kossel”, University of Rostock, 18147 Rostock, Germany

**Keywords:** Nerve ultrasound, Als, Mmn, Diagnosis, Nerve conduction studies

## Abstract

**Objective:**

To investigate diagnostic accuracy of a nerve ultrasound (US) protocol that is individualized to a patient’s clinical deficits for the differentiation of amyotrophic lateral sclerosis with predominant lower motoneuron disease (ALS/LMND) and multifocal motor neuropathy (MMN).

**Methods:**

Single-center, prospective, examiner-blinded, diagnostic study in two cohorts. Cohort I (model development): Convenience sample of subjects with ALS/LMND or MMN according to revised El-Escorial or EFNS guidelines. Cohort II (model validation): Consecutively recruited treatment-naïve subjects with suspected diagnosis of ALS/LMND or MMN.

Cutoffs for 28 different US values were determined by Receiver Operating Curve (ROC) in cohort I. Area Under The Curve (AUC) of US was compared to nerve conduction studies (NCS). Diagnostic accuracy of US protocols, individualized according to clinical deficits, was compared to former rigid non-individualized protocols and to random examination site selection in cohort II.

**Results:**

48 patients were recruited. In cohort I (28 patients), US had higher ROC AUCs than NCS, US 0.82 (0.12) (mean (standard deviation)), NCS (compound muscle action potential (CMAP) 0.60 (0.09), *p* < .001; two-sided *t*-test).

US models based on the nerve innervating the clinically most affected muscles had higher correct classification rates (CCRs, 93%) in cohort II than former rigid protocols (85% and 80%), or models with random measurement site selection (66% and 80%).

**Conclusions:**

Clinically guided US protocols for differentiation of ALS/LMND from MMN increase diagnostic accuracy when compared to clinically unguided protocols. They also require less measurements sites to achieve this accuracy.

**Supplementary Information:**

The online version contains supplementary material available at 10.1007/s00415-020-10323-6.

## Introduction

The differential diagnosis between amyotrophic lateral sclerosis with leading lower motoneuron disease presentation (ALS/LMND) and multifocal motor neuropathy (MMN) is of high prognostic and therapeutic importance while clinically sometimes demanding. Whereas ALS/LMND represents a fatal condition with no relevant disease-modifying treatment options, immune-modulating therapies are effective in MMN and increase strength and functional outcome [1].

Several studies have illustrated the value of nerve ultrasound (US) in this differential diagnostic question [2–4], reaching up to 100% sensitivity and 92% specificity for MMN in external validation study groups. The underlying diagnostic contrast is driven by a slight decrease in nerve cross-sectional area (CSA) in ALS/LMND on the one side [5], possibly due to accompanying axonal loss, and by a pronounced increase in CSA in MMN as a chronic inflammatory neuropathy on the other side [6].

Further studies support the clinical relevance of US assessments in patients with ALS/LMND or MMN. US measurements correlate with clinical scores like the Medical Research Council Sum Score (MRC-SS) and the Overall Disability Sum Score (ODSS) in MNN or with the MRC-SS and the revised ALS Functional Rating Scale (ALSFRS-R) in ALS/LMND [4]. In ALS, a decrease in CSA is limited to subtypes with lower motor neuron involvement and is not existent in primary lateral sclerosis (PLS) as a subtype without such involvement [7]. In addition, in ALS CSA correlates with cerebrospinal fluid (CSF) progranulin levels as a biochemical marker for axonal damage [8]. In MMN, CSA enlargements correlate with intraindividual longitudinal changes in clinical deficits and might therefore be suitable for the monitoring of therapeutic effects [6].

The above-mentioned diagnostic US models to differentiate MMN from ALS/LMND are based on standard examination protocols including 8 [4] or 10 [2] US measurements sites in different nerves, irrespective of where an individual patient shows his or her strongest clinical deficits. Such rigid and clinically unguided examination protocols are not only relatively laborious, but could have compromised diagnostic accuracy. The nerves included in the respective protocol are not necessarily the ones the strongest affected in an individual patient. Furthermore, repetitive measurements increase the risk of false-positive results.

The current prospective, monocenter, rater-blinded, disease-controlled diagnostic study tested the hypothesis that a clinically guided US examination of the clinically most strongly affected nerve only has equal or superior diagnostic power and is more efficient than the above-mentioned rigid and clinically unguided examination protocols for the differentiation of MMN from ALS/LMND. Former diagnostic US studies have been criticized for a potential spectrum bias due to study groups not representative of the diagnostic target population [9]. To address this, the hypothesis of the current study was tested in a prospectively recruited patient cohort without an established diagnosis of ALS/LMND or MMN, admitted for differential diagnosis of subacute acquired peripheral pure motor deficits of unknown etiology.

## Subjects and methods

### Study design, subjects and group assignment

This single-center, prospective, examiner-blinded cross-sectional diagnostic study was performed at the Department of Neurology, Technische Universität Dresden, Saxony, Germany. Two study cohorts were recruited between May 11th, 2017 and July 17th, 2018 (see Online Resource Figs 1 and 2 for study flow diagrams).

Cohort I consisted of patients with clinically established diagnoses and was used to develop diagnostic models and define US cutoff values. Cohort II consisted of patients admitted for the differential diagnosis of either ALS/LMND or MMN and served as an external validation group for the models.

Cohort I: Cohort I was conveniently recruited at our specialized outpatient clinic for patients with ALS and inflammatory neuropathies. Inclusion criteria were the diagnosis of ALS/LMND or MMN. For ALS, minimal diagnostic criterion was possible ALS according to revised El-Escorial criteria in combination with predominant LMN involvement [10]. Predominant LMN involvement was defined as presence of paresis without clinical or electrophysiological sings of upper motor neuron (UMN) involvement, such as increased muscle tone, spasticity, presence of pathological reflexes or increased central motor latency in magnetic evoked potentials. For MMN, minimal diagnostic criterion was a possible MMN according to the European Federation of Neurosciences/Peripheral Nerve Society (EFNS/PNS) guidelines [11].

Cohort II: Cohort II was a consecutive sample recruited from all patients admitted between May 11th, 2017 and July 17th, 2018 with a suspected diagnosis of ALS/LMND or MMN. For study eligibility, this differential diagnostic focus had to be reconfirmed by the attending senior physician during initial clinical ward rounds.

Exclusion criterion was inability to follow the study protocol (e.g., due to advanced impairment or severe dyspnea). All patients provided written informed consent. The study was approved by the institutional review board.

### Procedures

Basic demographic data were recorded (age, sex, disease duration and time since diagnosis in months). For clinical signs and symptoms, the following scales were employed: Medical Research Council Sum Score (MRC-SS) [12], Modified Rankin Scale (mRS) [13], EuroQol (EQ-5D-5L) [14] revised ALS Functional Rating Scale (ALSFRS-R, ALS/LMND only) [15], Overall Disability Sum Score (ODSS, MMN only) [13], Rasch-built Overall Disability Scale for Multifocal motor neuropathy (MMN-RODS, MMN only) [14]. Grip force was assessed with a dynamometer (Jamar hydraulic handgrip dynamometer, Lafayette Instrument Inc., Loughborough, UK).

Motor and sensory NCS (motor and sensory nerve conduction velocities (CVs), compound muscle action potentials (CMAPs)) of median, ulnar, radial, tibial, peroneal and sural nerves and f-responses of the median, ulnar and tibial nerves were obtained on a Keypoint electrophysiological system, Software Version 3.5 (Medtronic, Meerbusch, Germany) by experienced examiners (C.L., J.D.) blinded to group assignment. In addition to the measurement values, the presence of motor conduction blocks (defined as a reduction of > 50% in amplitude or area under the curve (AUC)) was determined. Patients were examined unilaterally on the clinically more affected side.

Both transverse and longitudinal US scans were obtained in 12 nerves/nerve roots at 14 different sites to record CSA and diameter (nerve/nerve roots examined: C5,6,7, superior trunk, vagal, radial, ulnar, median, sciatic, peroneal, tibial and sural nerve). Three images were taken at each measurement site and the mean calculated. Measurement sites were chosen according to a highly standardized and reproducible study protocol proposed by Zaidman and co-workers [16]. US machines used were: Aplio MX, linear transducer, 8–18 MHz (Toshiba, Neuss, Germany) and MyLab Five, linear transducer, 10–18 MHz (Esaote Biomedica, Cologne, Germany). Examinations were performed by two experienced investigators (R.W., K.F.L.), blinded to group assignment.

### Statistical analysis and diagnostic models

Statistical significance was defined by *p* < 0.05. If not otherwise stated, numbers given are mean and standard deviation (SD)*,* and the *p* value derived from a two-sided *t*-test. Normal distribution of continuous data was tested with Kolmogorov–Smirnov test. If data were normally distributed, two-sided *t*-tests were used for all two-group comparisons, otherwise a univariate ANOVA with a post hoc two-sided *t*-test. If data were not normally distributed or ordinal, a Mann–Whitney-*U*-test or a Kruskal–Wallis test with a post-hoc Mann–Whitney-*U*-test was used. All post hoc tests were Bonferroni-adjusted for α-inflation. For categorical data, Fisher’s exact test was used, in case of three groups with a Freeman-Halton extension. Correlations were assessed by Pearson’s correlation coefficient.

Receiver operating curve (ROC) analysis was performed to compare the area under the curve (AUC) for all single measurements obtained by US and NCS and to obtain cutoff values for all US measurements. Cutoff values were chosen from the respective ROC analysis in cohort I as defined by the highest Youden-index, or a minimum specificity of ≥ 80%, ≥ 90%, ≥ 95% or 100%.

The nerve/nerve root with the most heavily affected downstream muscle functions was determined by MRC-SS-based clinical testing of single muscle functions that could be unequivocally assigned to a single nerve; in case of that several muscle functions could be attributed to one nerve, the mean was considered (for muscle-nerve assignment, see Table 1). Functions that could be assigned to more than one nerve were not considered. Nerve roots naturally shared single muscle functions with peripheral nerves and were differentiated as far as possible based on single muscle functions (Table 1).

**Table 1:** Muscle functions as attributed to nerves/nerve roots and distribution of most heavily affected nerves/nerve roots according to muscle function testing, divided by study groups

Nerve/Nerve root	Attributed muscle functions	Cohort I		Cohort II		
		ALS n = 20	MMN n = 8	ALS n = 10	Other n = 5	MMN n = 5
C5/C6 root, superior trunk	Shoulder abduction, C5 (C6) root Ellbow flexion, (C5) C6 root Ellbow flexion with semi-pronated forearm, (C5) C6 root (radial nerve)	5 (25%)	2 (25%)	5 (50%)	2 (40%)	1 (20%)
C7 root	Ellbow extension, (C6) C7 root (radial nerve) Finger extension, C7 root (radial nerve)	2 (10%)^a^	4 (50%)^a^	2 (20%)	0	0
Radial nerve	Ellbow flexion with semi-pronated forearm Ellbow extension Wrist extension Finger extension Thumb radial abduction	3 (15%)^b^	5 (62%)^b^	2 (20%)	0	0
Ulnar nerve	Finger flexion at DIP joints IV/V Finger abduction Thumb adduction	1 (5%)	1 (13%)	0	0	2 (40%)
Median nerve	Pronation of forearm with extended elbow Finger flexion at PIP joints II/III/IV/V Thumb flexion at IP joint Thumb palmar abduction	7 (35%)	2 (25%)	2 (20%)	2 (40%)	1 (20%)
Sciatic nerve	Knee flexion	1 (5%)	0	0	1 (20%)	0
Tibial nerve	Foot plantar flexion Foot inversion Toe flexion	1 (5%)	0	0	1 (20%)	0
Peroneal nerve	Foot dorsal extension Foot eversion Toe extension	6 (30%)	0	4 (40%)	1 (20%)	1 (20%)

Table shows the attribution of muscle functions to specific nerves/nerve roots as the basis for the clinical decision on the nervous structure in an individual patient with the heaviest associated clinical deficit. With the exception of nerve roots, only those single muscle functions were considered that could be unequivocally attributed to a specific nervous structure

Numbers (percentages) given indicate how often the respective nerve/nerve root was associated with the heaviest clinical deficit according to single muscle testing by MRC-SS separate for all study groups. Summed percentages surpass 100%, because several patients had the heaviest muscle deficits in several single muscles, attributed to different nerves

In cohort I only, C7 nerve root (^a^*p* = .038) and radial nerve (^b^*p* = .022, Fisher’s exact test for both) were significantly more often associated with the most pronounced clinical deficit in MMN. In addition, in both cohorts there was a trend for the peroneal nerve to be more often selected in ALS/LMND than in MMN patients

*ALS* amyotrophic lateral sclerosis, *MMN* multifocal motor neuropathy, *n* number*, DIP* distal interphalangeal, *PIP* proximal interphalangeal, *IP* interphalangeal

In case of that the above-mentioned clinical testing procedure resulted into two or more nerves/nerve roots being affected to the same extent, the following four strategies were applied to define the single nerve/nerve root to be included into an individualized US testing protocol. All strategies were systematically compared for their diagnostic performance (for details, see Fig. 2):

1. In case of that the heaviest single muscle deficit could either be attributed to a consecutive proximal or distal nervous structure (e.g., C5 root vs. superior trunk; C6 root vs. superior trunk; C7 root vs. radial nerve; sciatic nerve vs. peroneal nerve or sciatic nerve vs. tibial nerve): Systematic comparison of protocols that either included the more proximal or the more distal structure (more proximal: models 1, 2, 5 & 6 vs. more distal: models 3, 4, 7 & 8, Fig. 2).
2. Systematic comparison of protocols that did or did not consider nerve roots in the examination site determination process (with cervical roots: models 2, 4, 6 & 8 vs. without cervical roots: models 1, 3, 5 & 7, Fig. 2).
3. In case of that equal and heaviest muscle deficits were present in two distinct nonconsecutive nervous structures: Decision for the nerve/nerve root easier to assess by US according to the following order of increasing difficulty: arm < leg < neck; ulnar nerve < median nerve < radial nerve; peroneal nerve < tibial nerve; C5 root < C6 root < C7 root; superior trunk < C7 root (applied to all models 1–8, Fig. 2).
4. Comparison of models that consider both CSA and diameter or CSA only (Both CSA and diameter: models 1, 2, 3, & 4 vs. CSA only: models 5, 6, 7 & 8).

The application of these four selection rules resulted in eight different models (Fig. 2). Based on the cutoff values determined by ROC analysis in cohort I (Table 2), the diagnostic performance of the eight different models was systematically assessed in both cohorts I & II (Fig. 3). In nerves where two measurement sites were assessed (median and ulnar nerve), both sites were considered and scored as pathological if either one of the two exceeded the respective cutoffs. In addition, the diagnostic performance of these eight different models guided by the distribution of clinical deficits was compared to formerly published clinically-unguided protocols [4, 17] (models 11 & 12), and to random clinically unguided examination site selection (models 9 & 10) (Fig. 3).

**Table 2:** Nerve-specific cutoff values (CSA & diameter) with a minimum specificity of 95% as determined by group-based ROC analysis in cohort I

CSA	ROC AUC (95% CI)	Cutoff value 95% specificity for MMN (mm^2^)
Radial nerve upper arm	1.00 (1.00–1.00)	≥ 4.50
Ulnar nerve upper arm	0.86 (0.70–1.00)	≥ 6.68
Ulnar nerve forearm	0.85 (0.70–1.00)	≥ 5.63
Median nerve upper arm	0.83 (0.65–1.00)	≥ 9.33
Median nerve forearm	0.83 (0.61–1.00)	≥ 7.12
Tibial nerve lower leg	0.73 (0.54–0.93)	≥ 9.37
Sciatic nerve upper leg	0.71 (0.48–0.95)	≥ 50.38
Peroneal nerve upper leg	0.40 (0.10–0.70)	≥ 7.60

Diameter	ROC AUC (95% CI)	Cutoff value 95% specificity for MMN (mm)
Radial nerve upper arm	0.97 (0.92–1.00)	≥ 1.87
Sciatic nerve upper leg	0.95 (0.87–1.00)	≥ 6.2
Median nerve forearm	0.88 (0.70–1.00)	≥ 2.62
Median nerve upper arm	0.82 (0.64–1.00)	≥ 3.00
Ulnar nerve upper arm	0.80 (0.64–0.97)	≥ 3.17
Ulnar nerve forearm	0.73 (0.53–0.93)	≥ 2.22
Tibial nerve lower leg	0.70 (0.49–0.92)	≥ 3.15
Peroneal nerve upper leg	0.67 (0.41–0.93)	≥ 2.63

Table shows cutoff values for CSA and diameter US measurement values with a minimum specificity of 95% in group ROC analysis in cohort I. These cutoff values were applied in the clinically guided individualized US protocols according to the decision flow diagram in Fig. 2. This procedure resulted in the diagnostic accuracies as given in Fig. 3. AUCs with a lower border of the 95% confidence interval of  ≤ 0.50 were not significant (*p*  ≥ .50).

*US* ultrasound, *CSA* cross-sectional area, *ROC* receiver operating curve, *AUC* area under the curve, *95% CI* 95% confidence interval

All analyses were performed with SPSS Version 25 (IBM, Ehningen, Germany).

## Results

48 patients were included, of which 28 allocated to cohort I for diagnostic model development and 20 to cohort II for subsequent external model testing.

Cohort I was conveniently recruited and comprised 20 patients with ALS/LMND and 8 with MMN. For external testing in a study cohort representative of a realistic diagnostic target population, cohort II consisted of 20 patients with a clinically suspected diagnosis of ALS/LMND or MMN, consecutively recruited from all 601 patients admitted to the general neurology ward of the study center between May 11th, 2017 and July 17th, 2018 (details in Table 3). After comprehensive routine diagnostic procedures (US as index test not considered), the 20 patients of cohort II received the following diagnoses: ALS/LMND 13, MMN 5, other diagnoses 4 (multifocal acquired demyelinating sensory and motor neuropathy (MADSAM), chronic inflammatory demyelinating polyneuropathy (CIDP), inclusion body myositis and multiple sclerosis) (Table 3). Of note, paraclinical findings considered to be supportive of MMN (GM1 antibodies, NCS conduction blocks) only offered limited group separation, with GM1 antibodies in up to 50% of ALS/LMND patients and NCS conduction blocks in only up to 63% of MMN patients (Table 3).

**Table 3:** Demographics, paraclinical findings and symptoms scores

	Cohort I			Cohort II				Group differences
	ALS (n = 20)	MMN (n = 8)	Test	ALS (n = 11)	MMN (n = 5)	Other (n = 4)	Test	
Demographics								
Age, mean (SD), years	62.90 (10.68)	64.00 (10.03)	*t*(26) = -0.25, *p* = .804^a^	66.90 (7.46)	49.80 (23.12)	60.20 (24.69)	*F*(2,17) = 1.64, *p* = .224^b^	
Female sex, n (%)	7 (35%)	1 (13%)	*p* = .371^e^	4 (37%)	1 (20%)	1 (25%)	*p* = 1.000^f^	
Symptom onset, median (IQR), months	18.50 (41.00)	87.00 (159.00)	*U* = 20.00, *p* = .001^c^	23.00 (19.00)	55.00 (272.00)	64.00 (123.00)	*H*(2) = 2.65, *p* = .266^d^	
Diagnosis time point, mean (SD), months	9.00 (19.00)	74.50 (84.00)	*U* = 8.00, *p* < .001^c^	n.a	n.a	n.a	n.a	
Diagnosis								
	Clin. definite 5 (25%)	Definite 5 (63%)		Clin. definite 0 (0%)	Definite 3 (60%)			
	Clin. probable 5 (25%)	Probable 3 (37%)		Clin. probable 0 (0%)	Probable 2 (40%)			
	Clin. probable – lab. supp. 10 (50%)			Clin. probable – lab. supp. 5 (42%)				
	Clin. possible 0 (0%)			Clin. possible 7 (58%)				
Paraclinical findings								
GM1-IgM-AB for those tested, n (%)	4 (50%)	1 (9%)	*p* = .111^e^	0 (0%)	2 (40%)	0 (0%)	*p* = .133^f^	
Definite motor CB for those tested, n (%)	0 (0%)	5 (63%)	*p* = .002^e^	0 (0%)	3 (60%)	0 (0%)	*p* = .017^f^	MMN > ALS; MMN > other
Clinical findings								
Handgrip force, mean (SD), NM	11.50 (8.40)	22.50 (8.21)	*t*(26) = – 3.15, *p* = .004^a^	15.00 (8.03)	22.4 (7.64)	16.60 (9.40)	*F*(2,17) = 1.35, *p* = .285^b^	
ALSFRS-R score, median (IQR)	33.0 (13.0)	n.a	n.a	38.0 (11.0)	n.a	n.a	n.a	
ODSS, median (IQR)	n.a.	3.0 (3.0)	n.a	n.a	1.0 (3.0)	n.a	n.a	
MMN-RODS, median (IQR)	n.a	49.5 (20.0)	n.a	n.a	49.0 (7.0)	n.a	n.a	
mRS, median (IQR)	3.0 (2.0)	1.5 (2.0)	*U* = 37.50, *p* = .028^c^	3.0 (2.0)	1.0 (1.0)	2.0 (2.0)	*H*(2) = 7.45, *p* = .024^d^	MMN < ALS
EQ-5D-5L index, median (IQR)	0.51 (0.65)	0.91 (0.41)	*U* = 35.50, *p* = .021^c^	0.5 (0.6)	1.0 (0.3)	0.4 (0.6)	*H*(2) = 6.02, *p* = .049^d^	ALS < MMN
EQ-5D–5L VAS, median (IQR)	50.0 (35.0)	60.0 (36.0)	*U* = 72.00 *p* = .709 ^c^	50.0 (41.0)	70.0 (13.0)	80.0 (43.0)	*H*(2) = 3.66, *p* = .160^d^	
MRC-SS, median (IQR)	46.00 (21.00)	55.50 (6.50)	*U* = 28.50, *p* = .007^c^	43.50 (13.00)	58.00 (10.00)	50.00 (21.00)	*H*(2) = 6.95, *p* = .031^d^	ALS < MMN

Data are displayed as mean (SD) for normally distributed data or median (IQR) for continuous data without normal distribution and ordinal data. For categorical data, numbers (percentages) are given. Diagnostic level is given for ALS/LMND according to revised El-Escorial criteria [10], and for MMN according to EFNS/PNS guidelines. Other diagnoses in cohort II were: MADSAM, CIDP, inclusion body myositis, and multiple sclerosis (one each)

Normality was assessed by Kolmogorov–Smirnov test. For continuous data with normal distribution, two-sided *t* test^a^, or ANOVA^b^ with post hoc two-sided *t* test was performed. For continuous data without normal distribution and for ordinal data, Mann–Whitney *U* test^c^, or Kruskal–Wallis test^d^ with post-hoc Mann–Whitney *U* test was performed. For categorical data, Fisher’s exact test^e^ was performed, in case of three groups with a Freeman-Halton extension^f^. Significant group differences were assumed if *p* < .05 for all tests (with Bonferroni adjustment for α inflation where applicable)

*ALS* amyotrophic lateral sclerosis, *MMN* multifocal motor neuropathy, *n* number, *SD* standard deviation, *IQR* interquartile range, *clin*. clinically, *lab*. laboratory, *supp*. supported, *AB* antibody, *CB* conduction block, *NM* newton meter, *ALSFRS-R* revised ALS functional rating scale, *ODSS* overall disability sum score, MMN-*RODS* Rasch-built overall disability scale for MMN, *mRS* Modified Ranking Scale, *EQ-5D-5L* EuroQol value set with five levels of severity for each of the five dimensions, *VAS* visual analog scale, *MRC-SS* Medical Research Council sum score, *n.a.* not applicable

### Cutoff value selection by ROC analysis

**Fig. 1:**
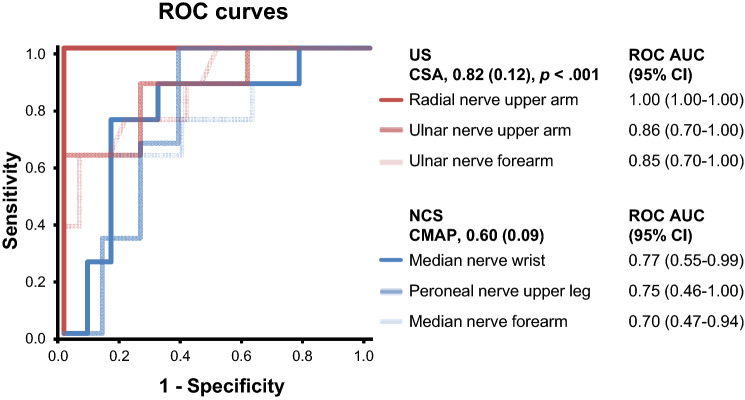
ROC curves of best ultrasound (US) and nerve conduction studies (NCS) measurement values ROC curves of the three best US measurement values (as defined by highest AUC) as considered in the models with the best diagnostic performance (models no. 5 and 7, Fig. 3) are shown (red curves). In comparison, the three best values from NCS (all CMAPs, blue curves) had lower AUCs. AUCs with a lower border of the 95% confidence interval of ≤ 0.50 were not significant (*p* ≥ .50). Other classes of NCS values than CMAPs had even lower AUCs (not shown). Mean AUC of all US values (0.82 (0.12)) was significantly higher than mean AUC (0.60 (0.09)) of all CMAP values at the best group of NCS values (*p* < .001; two-sided *t*-test). Abbreviations: *US* ultrasound, *NCS* nerve conduction studies, *CSA* cross-sectional area, *ROC* receiver operating curve, *AUC* area under the curve, *95%CI* 95% confidence interval

Of 28 different US measurements in 12 different nerves/nerve roots, 21 (75%) showed significant group differences in cohort I with lower CSA/diameter in ALS/LMND than in MMN (Online Resource Table 3). ROC analyses in cohort I showed superior discrimination for US than for NCS between ALS/LMND and MMN (Fig. 1, NCS CMAP, AUC (mean (stdv.)), 0.60 (0.09); US CSA/diameter, 0.82 (0.12), *p* < 0.001). All other NCS parameters (sensory and motor CVs, F responses) had even worse AUC than CMAPs (data not shown). Cutoff values to be used in the subsequent clinically guided diagnostic US models were determined for each single nerve by the ROC analysis in cohort I. Cutoff values with a minimum specificity of 80%, 90%, 95% and 100% or the highest Youden-Index were selected and systematically compared for their diagnostic performance in clinically guided US examination protocols (for cutoff values, see Table 3, and not shown).

### Determination of most heavily affected nervous structure by clinical deficits and examination site selection process

The selection process for the most heavily affected nerve/cervical root as the basis for measurement site selection in a clinically guided US protocol is given in Table 1. In cohort I, C7-cervical root or radial nerve-dependent muscle functions were significantly more often primarily affected in MMN patients (50% vs. 10% and 62% vs. 15%, respectively, *p* < 0.05 for both). In both cohorts, there was a trend for leg nerves to be more often primarily affected in ALS/LMND than in MMN patients (*p* > 0.05 for all).

The diagnostic potential of a clinically guided US examination protocol relies on a correlation between clinical deficits and US measurement values. Such a correlation was found for MMN patients only (*r*(38) =  – 0.38, *p* = 0.009, all nerves, clinical deficits assessed by MRC-SS, correlated with *z*-transformed US values). This correlation was stronger if only the clinically most affected nerve of each patient was taken (*r*(6) =  – 0.65, *p* = 0.042), supporting the rationale of choosing the clinically most affected nerve as the site of US examination.

As to be expected, in many cases clinical deficits did not unequivocally identify one nervous structure. Single muscle testing in an individual patient often revealed muscle functions of two different nerves as being affected to the same extent. In case of distal muscle functions deficits could be attributed to consecutive nerves (e.g., sciatic vs. peroneal nerve for dorsal foot extension or C7 root vs. radial nerve for elbow extension).

**Fig. 2:**
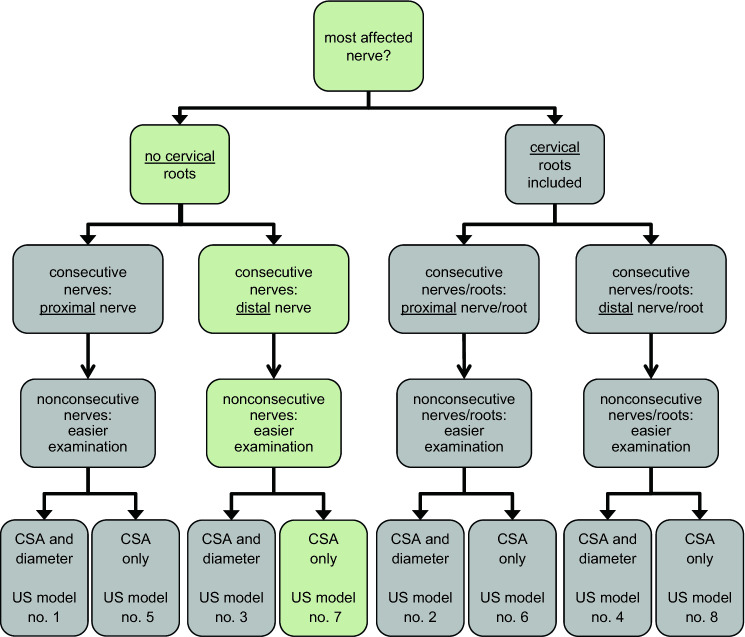
Flow chart of different single nerve/nerve root selection strategies compared. Flowchart shows the different strategies assessed for the determination of the nerve/nerve root to be included into an individualized US measurement protocol based on the results of clinical testing of motor deficits in an individual patient. The model suggested for diagnostic application is shown in shaded green (model no. 7). This model is given preference because models without cervical roots had slightly better diagnostic performance (see Fig. 3) and distal nervous structures and CSA values were considered to be more easily measurable by US than proximal nervous structures and diameter measurements. However, diagnostic performance was stable or only slightly lower (models with cervical roots considered, see Fig. 3), independent of the specific measurement site selection strategy applied. The following models were compared: Models with (models no. 1, 3, 5 & 7) or without (models no. 2, 4, 6 & 8) consideration of cervical roots; Models with the consideration of the more proximal (models no. 1, 2, 5 & 6) or more distal (models no. 3, 4, 7 & 8) nerve/nerve root in the case of the most pronounced deficit downstream to consecutive nervous structures. If two nonconsecutive nervous structures were affected to the same extent, the following was applied (to all models no. 1–8): selection of the nerve/nerve root easier to examine based on the following rules: (in order of increasing difficulty): (1) arm < leg < neck; (2) ulnar nerve < median nerve < radial nerve; (3) peroneal nerve < tibial nerve; (4) C5 root < C6 root < C7 root; (5) superior trunk < C7 root. Abbreviations: *CSA* cross-sectional area, *no.* number, *US* ultrasound

Thus, different selection strategies can be chosen for the nervous structure to be examined by US based on individual clinical deficit distribution and were systematically compared for their diagnostic performance (for selection flow diagram and resulting models no. 1–8, see Fig. 2, for nerves finally selected according to each model, see Online Resource Table 3).

### Diagnostic performance of clinically guided US protocols in comparison with clinically unguided former protocols and random examination site selection

**Fig. 3:**
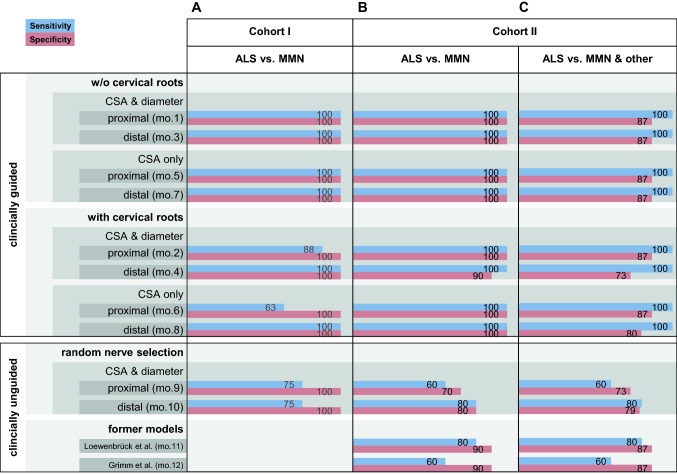
Diagnostic performance of clinically guided US examination protocols based on different selection strategies in comparison to clinically unguided protocols and random examination site selection. Figure shows diagnostic performance as expressed by sensitivity and specificity of all eight different strategies (models no. 1–8) for a clinically guided selection of a single nerve/nerve root to be examined by ultrasound, and compares it with formerly published models with rigid clinically unguided examination protocols that are based on the assessment of 8 or 10 different measurement values (models no. 11 & 12) [4, 17]. Additionally, the performance of all models is compared to random examination site selection (models no. 9 & 10). It is indicated whether or not cervical roots were considered (models no. 2,4,6 & 8 vs. models no. 1,3,5 & 7), and whether or not diameter values were considered in addition to CSA values (models no. 1,2,3 & 4 vs. models no. 5,6,7 & 8). For details concerning the different strategies to select the examination site in the clinically guided protocols see Fig. 1. Clinically guided models no. 1–8 and models based on random examination site selection (models no. 9 & 10) considered the cutoffs with a minimum specificity of 95% as derived from the group ROC analysis in cohort I (for cutoff values, see Table 2). In the formerly published models (models no. 11 & 12) the respective model-specific cutoff values were applied as published before [4, 17]. A. shows the diagnostic performance of all models when applied to study cohort I. B. shows the diagnostic performance when applied to cohort II and patients with final diagnoses other than ALS/LMND or MNN were excluded. C. shows the diagnostic performance when patients with final diagnoses other than ALS/LMND or MMN were not excluded from analysis. If patients with other diagnoses were not excluded from cohort II, two of those were classified as MMN. These two patients had other chronic inflammatory neuropathies as final diagnoses because of a more important sensory involvement than initially considered at study inclusion. CSA cross-sectional area, mo. model, ALS amyotrophic lateral sclerosis, MMN multifocal motor neuropathy, no. number

Starting with a minimum specificity of 95% of the cutoff value selected by group ROC analysis in cohort I, up to 100% specificity and sensitivity were achieved when applied to the strongest affected nerve (Fig. 3). Thus, these cutoff values were selected for further usage and comparison of diagnostic models (Table 2).

The accuracy of the clinically guided models based on the different selection strategies (Fig. 2, models 1–8) was compared to models based on clinically unguided random measurement value selection (Fig. 3, models 9 & 10) and to formerly published rigid examination protocols including 8 and 10 US values (Fig. 3, models 11 & 12), respectively [2, 4].

Overall, clinically guided US protocols, based on the clinically most affected nerve only, had superior diagnostic accuracy in both cohorts (Fig. 3, models 1–8), no matter whether compared to former rigid examination protocols or to models with random nerve selection. Among the clinically guided models, the ones including nerve root measurement sites (Fig. 3, models 2, 4, 6 & 8) had a tendency to a lower diagnostic accuracy than those with measurement values form peripheral nerves only (Fig. 3, models 1, 3, 5 & 7). In- or exclusion of diameter measurements (Fig. 3, models 1 & 2 vs. models 5 & 7) had no effect on accuracy, as well as the preference of proximal or distal measurement points in the case of consecutive nerves (Fig. 3, models 1 & 5 vs. models 3 & 7). The models with the highest diagnostic accuracy (Fig. 3, models 1,3,5 & 7) reached 100% correct classification rate for MMN in both cohorts, if patients with other diagnoses than ALS/LMND or MMN were excluded from the validation cohort II. If patients with other diagnoses were included, the specificity of these models dropped to 87%, due to the misclassification of two subjects as MMN. These two subjects both had the final diagnosis of other acquired inflammatory demyelinating neuropathies (MADSAM or CIDP), in both cases due to a stronger sensory involvement than recognized during initial clinical evaluation at study inclusion.

## Discussion

All diagnostic US models suggested so far for different neuropathies and/or ALS/LMND rely on rigid examination protocols that are not individualized according to the distribution of clinical deficits of the respective patient. Such rigid protocols do not consider the often uneven and in many cases even focused distribution of clinical deficits, even though US of nerval structures in an individual patient’s clinical focus can be expected to be crucial for the highest diagnostic contrast. If such rigid examination protocols are too condensed and consequently risk leaving out the clinical focus, they could generate false-negative results and lower sensitivity. If such rigid examination protocols are extensive or even comprehensive for all accessible nerval structures, this can impede broad clinical implementation and generate false-positive results and lower specificity due to repetitive testing and due to the inclusion of technically challenging anatomic sites. To our knowledge, this is the first US study in both neuropathies and ALS/LMND to abandon rigid examination protocols in favor for a clinically guided flexible examination protocol.

The results support both hypotheses that were the rationale of the current study:

Not only offered the clinically guided approach higher diagnostic accuracy in comparison to the rigid examination protocols formerly published by two independent research groups [2, 4], reaching CCRs of up to 100% instead of 87% or 81% in cohort II (Fig. 3), but also was it more time-efficient. The inclusion of only nerve upstream of the most heavily affected muscle functions sufficed, instead of the 8 or 10 values to be measured for the former rigid examination protocols.

This increase in diagnostic accuracy of the clinically guided US protocols appears to be driven by the correlation of US changes and clinical deficits in MMN patients, whereas no such correlation could be found in ALS/LMND patients in our study group. This correlation in MMN patients is stronger in the clinically most affected nerve than in all nerves, further supporting the rationale to select the clinically most affected nerve for US examination.

Of the four models with equal and superior diagnostic accuracy (Fig. 3, models 1, 3, 5 & 7), the most convenient model (Fig. 3, model 7) could be suggested for routine diagnostic application; Peripheral nerves are mostly more easily accessible by US than proximal ones (Fig. 3, model 5), and CSA are more conventionally used than diameter measurements (Fig. 3, models 1 & 3). Thus, a workflow for practical clinical application of model 7 could be: 1. Decide on the clinically most affected nerve by single muscle function testing according to MRC-SS. 2. If there are two different nerves clinically equally affected, decide for the one easier to examine by US. If there are consecutive nerves responsible for the respective clinical deficit, decide for the more distal nerve. 3. Measure CSA values in that one nerve only and score the results according to the cutoff values given in Table 2.

In addition, several sensitivity analyses support the robustness of this approach: The same high accuracy was obtained whether or not diameter measurements in addition to CSA were considered (Fig. 3, models 1 & 3 vs. models 5 & 7), or whether proximal or distal consecutive nerves were considered (Fig. 3, models 1 & 5 vs. models 3 & 7). The usefulness of a clinically guided measurement site selection was further supported by the inferiority of models based on random selection (Fig. 3, models 9 & 10, CCRs in cohort II: 66% and 79%, respectively).

The current results are consistent with results of former descriptive or diagnostic studies on ALS/LMND and/or MMN: as to be expected based on known differences in clinical presentation [18, 19], the clinically most affected muscle functions were more often caudal in ALS/LMND than in MMN, such as foot or toe extension. As in former diagnostic US and NCS studies on the differentiation of ALS/LMND from MMN, ROC analyses showed a lower discriminatory power for NCS than for US (Fig. 1) [4].

Concerning the diagnostic potential of US measurements from cervical nerve roots in the contrast between ALS/LMND and MMN, former studies have heterogeneous results: Two former studies from independent study groups found a lower diagnostic potential of cervical nerve roots than peripheral nerves in the differentiation of ALS/LMND from MMN [4], or in the differentiation of ALS subtypes [20]. However, results of another study on the broader question of the differentiation of sensorimotor chronic inflammatory neuropathies from other forms of acquired neuropathies advise the cautious interpretation of the diagnostic potential of nerve roots [21]: The latter study achieved a similarly high diagnostic accuracy as the current study and included cervical roots in the measurement protocol. The diagnostic strategy differed substantially from the current one: The study was based on a rigid examination protocol including bilateral measurements from median nerves and cervical roots and relied on very rigid cutoff values of 99% specificity. The reason for these mixed results on the role of cervical roots remains speculative and warrants further investigation: Contributors could be differences in measurement protocol and strategies for cutoff value selection, an increased measurement error due to the technically more challenging examination of nerve roots compared to peripheral nerves, or differences of the individual distribution of morphologic changes in relatively small study cohorts.

In conclusion, the results of the current study suggest that a clinically guided US examination protocol, based on the assessment of the clinically strongest affected nerve only, could be both accurate and efficient in the differentiation of ALS/LMND from MMN. In addition, implementation is technically relatively simple, since the acquisition of easy-to-obtain CSA measurements from distal nerves obtained the same or higher diagnostic accuracy than other measurement site selection strategies compared.

Our study has several limitations. First, it was monocenter with a restricted number of participants. Even though patients were transferred from all district hospitals in the City of Dresden (> 530,000 inhabitants) and from nearby regions of Eastern Saxony (> 1,000,000 inhabitants), only 20 patients could be recruited for cohort II. Thus, the reported diagnostic accuracy has to be regarded with caution. Results of the current study need to be reconfirmed in larger cohorts and by independent examiners.

This and other studies point out the importance to incorporate US into comprehensive and multimodal diagnostic workflows rather than to employ US as a separate complementary method. Whereas in the current study the distribution of clinical deficits was the premise for the US examination approach, in other studies NCS results were part of a defined multimodal diagnostic workflow and premise for a specific subsequent US examination [9, 22]. The promising potential of such defined sequential and multimodal diagnostic workflows should be further explored in future studies.

### Clinical implications

In the differentiation of ALS/LMND from MMN, US examination guided by the distribution of clinical deficits in an individual patient offers higher diagnostic accuracy and is more efficient in direct comparison to clinically unguided examination strategies. Whether this also applies for nerve US examination in other diagnostic questions beyond the current one warrants further exploration.

## Supplementary Information

Below is the link to the electronic supplementary material.

Supplementary file1 (DOCX 211 KB)

## Data Availability

Data/Materials are made available in this publication and in the supplementary materials. Additional data/materials are available from the authors upon request.
