# Supplementary material for: One nerve suffices: A clinically guided nerve ultrasound protocol for the differentiation of multifocal motor neuropathy (MMN) and amyotrophic lateral sclerosis (ALS)
Source: J Neurol. 2020 Dec 23;268(4):1495–507. doi: 10.1007/s00415-020-10323-6 (PMC7990818; doi:10.1007/s00415-020-10323-6)
Supplement: Supplementary file 1 — Supplementary file1 (DOCX 211 KB) [file 415_2020_10323_MOESM1_ESM.docx]

**Supplementary Materials**

**One nerve suffices: A clinically guided nerve ultrasound protocol for the differentiation of multifocal motor neuropathy (MMN) and amyotrophic lateral sclerosis (ALS)**

*Kai F. Loewenbrück, M.D.^1,2,*^, Robin Werner, M.D.^1^, René Günther, M.D.^1,2^, Markus Dittrich, M.D.^1,3^, Robert Klingenberger, M.D.^1^, Heinz Reichmann, M.D.^1^, Ph.D., Alexander Storch, M.D.^4,5^, Andreas Hermann, M.D.,Ph.D.^5,6^*

*^1^ Department of Neurology, Technische Universität Dresden, 01307 Dresden, Germany*

*^2^ German Center for Neurodegenerative Diseases (DZNE), 01307 Dresden, Germany*

*^3^ Department of Neurology, Elblandkliniken, 01662 Meissen, Germany*

*^4^ Department of Neurology, University of Rostock, 18147 Rostock, Germany*

*^5^ German Center for Neurodegenerative Diseases (DZNE) Rostock/Greifswald, 18147 Rostock, Germany*

*^6^ Translational Neurodegeneration Section "Albrecht Kossel", Department of Neurology, University of Rostock, 18147 Rostock, Germany*

**Corresponding Author:**

Kai F. Loewenbrück, M.D.

Department of Neurology, Technische Universität Dresden; Fetscherstrasse 74, D-01307 Dresden, Germany & Deutsches Zentrum für Neurodegenerative Erkrankungen (DZNE) Dresden, Tatzberg 41, D-01307 Dresden, Germany, Phone: ++49-351-458-18518; Fax: ++49-351-458-4352, E-mail: kai.loewenbrueck@uniklinikum-dresden.de

**Supplementary Materials**

**Supplementary tables:**

**Table 1** Group comparisons of nerve ultrasound measurements for cohort I

**Table 2** Frequencies of most affected nerves in cohort I for different single nerve/nerve root selection strategies applied

**Table 3** Frequencies of most affected nerves in cohort II for different single nerve/nerve

root selection strategies applied

**Supplementary figures:**

**Fig. 1** StaRD study flow diagram for differential diagnosis of MMN and ALS in cohort I

**Fig. 2** StaRD study flow diagram for differential diagnosis of MMN and ALS in cohort II

**Table 1** Group comparisons of nerve ultrasound measurements for cohort I

| **Nerve** |  | **ALS** | **MMN** | **Test** |
| --- | --- | --- | --- | --- |
| **Median nerve** |  |  |  |  |
| **Forearm** |  |  |  |  |
|  | CSA (mm^2^) | 6.07 (1.21) | 8.65 (5.67) | *U* = 27.00, *p* = .006^b^ |
|  | Diameter (mm) | 2.20 (0.28) | 2.47 (0.49) | *t*(26) = -3.72, *p* = .001^a^ |
| **Upper arm** |  |  |  |  |
|  | CSA (mm^2^) | 7.88 (1.27) | 14.98 (11.83) | *U* = 26.00, *p* = .005^b^ |
|  | Diameter (mm) | 2.23 (0.74) | 2.98 (1.78) | *U* = 29.00, *p* = .008^b^ |
| **Ulnar nerve** |  |  |  |  |
| **Forearm** |  |  |  |  |
|  | CSA (mm^2^) | 4.85 (0.45) | 5.93 (2.41) | *U* = 23.00, *p* = .003^b^ |
|  | Diameter (mm) | 1.73 (0.30) | 2.05 (0.34) | *t*(26) = -2.44, *p* = .022^a^ |
| **Upper arm** |  |  |  |  |
|  | CSA (mm^2^) | 5.25 (1.34) | 7.35 (2.43) | *U* = 22.00, *p* = .002^b^ |
|  | Diameter (mm) | 1.95 (0.48) | 2.37 (0.32) | *U* = 31.50, *p* = .011^b^ |
| **Radial nerve** |  |  |  |  |
| **Upper arm** |  |  |  |  |
|  | CSA (mm^2^) | 3.48 (0.93) | 6.19 (2.13) | *U* = 0.00, *p* < .001^b^ |
|  | Diameter (mm) | 1.50 (0.43) | 2.10 (0.54) | *U* = 4.50, *p* < .001^b^ |
| **Vagal nerve** |  |  |  |  |
| **Neck** |  |  |  |  |
|  | CSA (mm^2^) | 1.73 (0.60) | 2.17 (0.43) | *U* = 34.00, *p* = .025^b^ |
|  | Diameter (mm) | 1.10 (0.30) | 1.40 (0.81) | *U* = 43.50, *p* = .084^b^ |
| **C5 root** |  |  |  |  |
| **Inter-scalene gap** | |  |  |  |
|  | CSA (mm^2^) | 5.13 (1.37) | 9.45 (7.89) | *U* = 19.50, *p* = .001^b^ |
|  | Diameter (mm) | 2.10 (0.40) | 2.97 (0.59) | *t*(25) = -4.48, *p* < .001^a^ |
| **C6 root** |  |  |  |  |
| **Inter-scalene gap** | |  |  |  |
|  | CSA (mm^2^) | 7.53 (2.10) | 11.23 (7.78) | *U* = 4.00, *p* < .001^b^ |
|  | Diameter (mm) | 3.26 (0.58) | 4.41 (1.03) | *t*(24) = -3.59, *p* = .001^a^ |
| **C7 root** |  |  |  |  |
| **Inter-scalene gap** | |  |  |  |
|  | CSA (mm^2^) | 9.31 (1.98) | 13.32 (2.81) | *t*(21) = -3.83, *p* = .001^a^ |
|  | Diameter (mm) | 3.03 (0.46) | 4.33 (0.69) | *t*(20) = -4.98, *p* < .001^a^ |
| **Superior trunk** | |  |  |  |
| **Supraclavicular fossa** | |  |  |  |
|  | CSA (mm^2^) | 18.27 (3.97) | 29.88 (12.88) | *U* = 3.00, *p* < .001^b^ |
|  | Diameter (mm) | 3.59 (0.74) | 5.39 (1.03) | *t*(22) = -4.69, *p* < .001^a^ |
| **Sciatic nerve** |  |  |  |  |
| **Upper leg** |  |  |  |  |
|  | CSA (mm^2^) | 39.76 (7.92) | 48.26 (14.40) | *t*(25) = -1.98, *p* = .058^a^ |
|  | Diameter (mm) | 4.55 (0.76) | 6.48 (1.04) | *t*(25) = -5.40, *p* < .001^a^ |
| **Peroneal nerve** |  |  |  |  |
| **Upper leg** |  |  |  |  |
|  | CSA (mm^2^) | 5.75 (0.88) | 5.03 (4.84) | *U* = 64.50, *p* = .438^b^ |
|  | Diameter (mm) | 2.19 (0.25) | 2.44 (0.56) | *t*(26) = -1.69 *p* = .103^a^ |
| **Tibial nerve** |  |  |  |  |
| **Lower Leg** |  |  |  |  |
|  | CSA (mm^2^) | 7.85 (2.20) | 8.75 (1.74) | *U* = 42.50, *p* = .055^b^ |
|  | Diameter (mm) | 2.51 (0.4) | 2.93 (0.64) | *t*(26) = -2.14, *p* = .042^a^ |
| **Sural nerve** |  |  |  |  |
| **Lower leg** |  |  |  |  |
|  | CSA (mm^2^) | 1.90 (0.43) | 1.95 (0.64) | *t*(26) = -0.24, *p* = .814^a^ |
|  | Diameter (mm) | 1.13 (0.22) | 1.13 (0.19) | *t*(26) = 0.06, *p* = .956^a^ |

Significant group differences were assumed if *p*<.050 for all tests

Normality was assesed by Kolmogorov-Smirnov test.

^a^ For data with normal distribution, table shows mean and standard deviation (SD), as well as a two-sided *t* test

^b^ For data without normal distribution, table shows median and interquartile range (IQR), as well as a Mann-Whitney *U* Test

*ALS* amyotrophic lateral sclerosis, *MMN* multifocal motor neuropathy, *CSA* cross-sectional area

**Table 2** Frequencies of most affected nerves in cohort I for different single nerve/nerve

root selection strategies applied

| **Cohort I (ALS 20, MMN 8)** | | | | | | | | | | |
| --- | --- | --- | --- | --- | --- | --- | --- | --- | --- | --- |
|  | **C5**  **Root** | **C6**  **root** | **C7**  **root** | **Superior**  **trunk** | **Median nerve** | **Ulnar nerve** | **Radial nerve** | **Sciatic nerve** | **Tibial nerve** | **Peroneal nerve** |
| **Model no. 1, CSA + diameter, no cervical roots, consecutive proximal** | | | | | | | | | | |
| **ALS** | 0 | 0 | 0 | 0 | 7 (35%) | 1 (5%) | 6 (30%) | 1 (5%) | 0 | 5 (25%) |
| **MMN** | 0 | 0 | 0 | 0 | 2 (25%) | 1 (13%) | 5 (63%) | 0 | 0 | 0 |
| **Model no. 2, CSA + diameter, with cervical roots, consecutive proximal** | | | | | | | | | | |
| **ALS** | 4 (20%) | 0 | **2 (10%)*** | 0 | 7 (35%) | 1 (5%) | 0 | 1 (5%) | 0 | 5 (25%) |
| **MMN** | 1 (13%) | 0 | **4 (50%)*** | 0 | 2 (25%) | 1 (13%) | 0 | 0 | 0 | 0 |
| **Model no. 3, CSA + diameter, no cervical roots, consecutive distal** | | | | | | | | | | |
| **ALS** | 0 | 0 | 0 | 0 | 7 (35%) | 1 (5%) | 6 (30%) | 0 | 0 | 6 (30%) |
| **MMN** | 0 | 0 | 0 | 0 | 2 (25%) | 1 (13%) | 5 (63%) | 0 | 0 | 0 |
| **Model no. 4, CSA + diameter, with cervical roots, consecutive distal** | | | | | | | | | | |
| **ALS** | 0 | 0 | 0 | 3 (15%) | 7 (35%) | 1 (5%) | **3 (15%)*** | 0 | 0 | 6 (30%) |
| **MMN** | 0 | 0 | 0 | 0 | 2 (25%) | 1 (13%) | **5 (63%)*** | 0 | 0 | 0 |
| **Model no. 5, CSA only, no cervical roots, consecutive proximal** | | | | | | | | | | |
| **ALS** | 0 | 0 | 0 | 0 | 7 (35%) | 1 (5%) | 6 (30%) | 1 (5%) | 0 | 5 (25%) |
| **MMN** | 0 | 0 | 0 | 0 | 2 (25%) | 1 (13%) | 5 (63%) | 0 | 0 | 0 |
| **Model no. 6, CSA only, with cervical roots, consecutive proximal** | | | | | | | | | | |
| **ALS** | 4 (20%) | 0 | **2 (10%)*** | 0 | 7 (35%) | 1 (5%) | 0 | 1 (5%) | 0 | 5 (25%) |
| **MMN** | 1 (13%) | 0 | **4 (50%)*** | 0 | 2 (25%) | 1 (13%) | 0 | 0 | 0 | 0 |
| **Model no. 7, CSA only, no cervical roots, consecutive distal** | | | | | | | | | | |
| **ALS** | 0 | 0 | 0 | 0 | 7 (35%) | 1 (5%) | 6 (30%) | 0 | 0 | 6 (30%) |
| **MMN** | 0 | 0 | 0 | 0 | 2 (25%) | 1 (13%) | 5 (63%) | 0 | 0 | 0 |
| **Model no. 8, CSA only, with cervical roots, consecutive distal** | | | | | | | | | | |
| **ALS** | 0 | 0 | 0 | 3 (15%) | 7 (35%) | 1 (5%) | **3 (15%)*** | 0 | 0 | 6 (30%) |
| **MMN** | 0 | 0 | 0 | 0 | 2 (25%) | 1 (13%) | **5 (63%)*** | 0 | 0 | 0 |
| **Model no. 9, Random nerve, CSA + diameter, with cervical roots** | | | | | | | | | | |
| **ALS** | 3 (15%) | 3 (15%) | 1 (5%) | 3 (15%) | 3 (15%) | 4 (20%) | 1 (5%) | **0*** | 1 (5%) | 1 (5%) |
| **MMN** | 0 | 1 (13%) | 0 | 0 | 1 (13%) | 1 (13%) | 1 (13%) | **3 (38%)*** | 1 (13%) | 0 |
| **Model no. 10, Random nerve, CSA+ diameter, no cervical roots** | | | | | | | | | | |
| **ALS** | 0 | 0 | 0 | 0 | 5 (25%) | 5 (25%) | 3 (15%) | 1 (5%) | 3 (15%) | 3 (15%) |
| **MMN** | 0 | 0 | 0 | 0 | 1 (13%) | 1 (13%) | 1 (13%) | 3 (38%) | 1 (13%) | 1 (13%) |

Table shows frequencies of most affected nerves according to different models in Cohort I. The absolute frequency is shown, as well as the relative frequency. For comparison of groups, Fisher’s Exact was performed, in case of three groups with Freeman-Halton extension. * Significant group differences appear in bold.

no. number, ALS amyotrophic lateral sclerosis, MMN multifocal motor neuropathy, CSA cross-sectional area

**Table 3** Frequencies of most affected nerves in cohort II for different single nerve/nerve

root selection strategies applied

| **Cohort II (ALS 10, others 5, MMN 5)** | | | | | | | | | | |
| --- | --- | --- | --- | --- | --- | --- | --- | --- | --- | --- |
|  | **C5**  **root** | **C6**  **root** | **C7**  **root** | **Superior**  **trunk** | **Median nerve** | **Ulnar nerve** | **Radial nerve** | **Sciatic nerve** | **Tibial nerve** | **Peroneal nerve** |
| **Model no. 1, CSA + diameter, no cervical roots, consecutive proximal** | | | | | | | | | | |
| **ALS** | 0 | 0 | 0 | 0 | 2 (20%) | 1 (10%) | 3 (30%) | 0 | 0 | 4 (40%) |
| **others** | 0 | 0 | 0 | 0 | 3 (60%) | 0 | 1 (20%) | 1 (20%) | 0 | 0 |
| **MMN** | 0 | 0 | 0 | 0 | 1 (20%) | 2 (40%) | 1 (20%) | 0 | 0 | 1 (20%) |
| **Model no. 2, CSA + diameter, with cervical roots, consecutive proximal** | | | | | | | | | | |
| **ALS** | 3 (30%) | 1 (10%) | 0 | 0 | 2 (20%) | 0 | 0 | 0 | 0 | 4 (40%) |
| **others** | 1 (20%) | 0 | 0 | 0 | 3 (60%) | 0 | 0 | 1 (20%) | 0 | 0 |
| **MMN** | 1 (20%) | 0 | 0 | 0 | 1 (20%) | 2 (40%) | 0 | 0 | 0 | 1 (20%) |
| **Model no. 3, CSA + diameter, no cervical roots, consecutive distal** | | | | | | | | | | |
| **ALS** | 0 | 0 | 0 | 0 | 2 (20%) | 1 (10%) | 3 (30%) | 0 | 0 | 4 (40%) |
| **others** | 0 | 0 | 0 | 0 | 3 (60%) | 0 | 1 (20%) | 0 | 0 | 1 (20%) |
| **MMN** | 0 | 0 | 0 | 0 | 1 (20%) | 2 (40%) | 1 (20%) | 0 | 0 | 1 (20%) |
| **Model no. 4, CSA + diameter, with cervical roots, consecutive distal** | | | | | | | | | | |
| **ALS** | 0 | 0 | 0 | 2 (20%) | 2 (20%) | 0 | 2 (20%) | 0 | 0 | 4 (40%) |
| **others** | 0 | 0 | 0 | 1 (20%) | 3 (60%) | 0 | 0 | 0 | 0 | 1 (20%) |
| **MMN** | 0 | 0 | 0 | 1 (20%) | 1 (20%) | 2 (40%) | 0 | 0 | 0 | 1 (20%) |
| **Model no. 5, CSA only, no cervical roots, consecutive proximal** | | | | | | | | | | |
| **ALS** | 0 | 0 | 0 | 0 | 2 (20%) | 1 (10%) | 3 (30%) | 0 | 0 | 4 (40%) |
| **others** | 0 | 0 | 0 | 0 | 3 (60%) | 0 | 1 (20%) | 1 (20%) | 0 | 0 |
| **MMN** | 0 | 0 | 0 | 0 | 1 (20%) | 2 (40%) | 1 (20%) | 0 | 0 | 1 (20%) |
| **Model no. 6, CSA only, with cervical roots, consecutive proximal** | | | | | | | | | | |
| **ALS** | 3 (30%) | 1 (10%) | 0 | 0 | 2 (20%) | 0 | 0 | 0 | 0 | 4 (40%) |
| **others** | 1 (20%) | 0 | 0 | 0 | 3 (60%) | 0 | 0 | 1 (20%) | 0 | 0 |
| **MMN** | 1 (20%) | 0 | 0 | 0 | 1 (20%) | 2 (40%) | 0 | 0 | 0 | 1 (20%) |
| **Model no. 7, CSA only, no cervical roots, consecutive distal** | | | | | | | | | | |
| **ALS** | 0 | 0 | 0 | 0 | 2 (20%) | 1 (10%) | 3 (30%) | 0 | 0 | 4 (40%) |
| **others** | 0 | 0 | 0 | 0 | 3 (60%) | 0 | 1 (20%) | 0 | 0 | 1 (20%) |
| **MMN** | 0 | 0 | 0 | 0 | 1 (20%) | 2 (40%) | 1 (20%) | 0 | 0 | 1 (20%) |
| **Model no. 8, CSA only, with cervical roots, consecutive distal** | | | | | | | | | | |
| **ALS** | 0 | 0 | 0 | 2 (20%) | 2 (20%) | 0 | 2 (20%) | 0 | 0 | 4 (40%) |
| **others** | 0 | 0 | 0 | 1 (20%) | 3 (60%) | 0 | 0 | 0 | 0 | 1 (20%) |
| **MMN** | 0 | 0 | 0 | 1 (20%) | 1 (20%) | 2 (40%) | 0 | 0 | 0 | 1 (20%) |
| **Model no. 9, Random nerve, CSA + diameter, with cervical roots** | | | | | | | | | | |
| **ALS** | 0 | 1 (10%) | 1 (10%) | 2 (20%) | 2 (20%) | 2 (20%) | 0 | 0 | 2 (20%) | 0 |
| **others** | 0 | 1 (20%) | 1 (20%) | 2 (40%) | 1 (20%) | 0 | 0 | 0 | 0 | 0 |
| **MMN** | 1 (20%) | 0 | 0 | 1 (20%) | 0 | 1 (20%) | 0 | 1 (20%) | 1 (20%) | 0 |
| **Model no. 10, Random nerve, CSA + diameter, no cervical roots** | | | | | | | | | | |
| **ALS** | 0 | 0 | 0 | 1 (10%) | 2 (20%) | 3 (30%) | 2 (20%) | 0 | 2 (20%) | 0 |
| **others** | 0 | 0 | 0 | 0 | 2 (40%) | 0 | 0 | 1 (20%) | 0 | 2 (40%) |
| **MMN** | 0 | 0 | 0 | 0 | 0 | 1 (20%) | 1 (20%) | 1 (20%) | 1(20%) | 1 (20%) |

Table shows frequencies of most affected nerves according to different models in Cohort I. The absolute frequency is shown, as well as the relative frequency. For comparison of groups, Fisher’s Exact was performed, in case of three groups with Freeman-Halton extension. * Significant group differences appear in bold.

no. number, ALS amyotrophic lateral sclerosis, MMN multifocal motor neuropathy, CSA cross-sectional area

**Fig. 1 StaRD study flow diagram for differential diagnosis of MMN and ALS in cohort I**

Study flow diagram for diagnosis of MMN in Cohort I. Diagram shows the US models with the best diagnostic accuracy and efficiency (models no. 5 and 7).

*n* number, *ALS* amyotrophic lateral sclerosis, *MMN* multifocal motor neuropathy, *US* ultrasound, *CCD* complex clinical diagnosis

**Fig. 2** StaRD study flow diagram for differential diagnosis of MMN and ALS in cohort II

Study flow diagram for diagnosis of MMN if patients with other diagnoses were not excluded from analysis.

Diagram shows the US models with the best diagnostic accuracy and efficiency (models no. 5 and 7).

*n* number, *ALS* amyotrophic lateral sclerosis, *MMN* multifocal motor neuropathy, *US* ultrasound, *CCD* complex clinical diagnosis
